# Supplementary material for: Traction force microscopy with optimized regularization and automated Bayesian parameter selection for comparing cells
Source: Sci Rep. 2019 Jan 24;9:539. doi: 10.1038/s41598-018-36896-x (PMC6345967; doi:10.1038/s41598-018-36896-x)
Supplement: Supplementary file 1 — Supplementary Material [file 41598_2018_36896_MOESM1_ESM.pdf]

Supplementary Material: Traction force microscopy with optimized regularization and automated Bayesian parameter selection for comparing cells

Yunfei Huang[1], Christoph Schell[2], Tobias B. Huber[4,5,6], Ahmet Nihat Şimşek[1], Nils Hersch[3], Rudolf Merkel[3], Gerhard Gompper[1], and Benedikt Sabass[1,*]

[1]Theoretical Soft Matter and Biophysics, Institute of Complex Systems-2 and Institute for Advanced Simulation, Forschungszentrum Juelich, D-52425, Juelich, Germany
[2]Institut für Klinische Pathologie, Universitätsklinikum Freiburg, D-79002, Freiburg, Germany
[3]Biomechanics, Institute of Complex Systems-7, Forschungszentrum Juelich, D-52425, Juelich, Germany
[4]Department of Medicine IV, Faculty of Medicine, Medical Center – University of Freiburg, Germany
[5]BIOSS Center for Biological Signalling Studies, Albert-Ludwigs-University Freiburg, Germany
[6]III. Department of Medicine, University Medical Center Hamburg-Eppendorf, Hamburg, Germany
[*]b.sabass@fz-juelich.de

ABSTRACT

This supplementary document provides details on the generation of test data, on the construction and solution of the optimization problems, on the choice of regularization parameters, and code. Figures contain further results on the performance of different TFM routines.

Contents

1 Fast calculation of the matrix M using the shift theorem in Fourier space 2
2 Proximal gradient methods for L1 regularization (PGL) and EN regularization (PGEN) 2
3 Bayesian compressive sensing using Laplace prior (BCSL) 3
4 Bayesian Lasso (BL) and Bayesian elastic net (BEN) 3
5 Analytical calculation of displacements around a circular traction patch 3
6 Implementation of the regularization routines 4
7 L1 regularization using Iterative Reweighted Least Squares (IRLS) 5
8 Implementation of Bayesian TFM routines 5
References 8

List of Figures

S1 Classical methods for selecting the regularization parameter . . . 9
S2 Additional error quantification for the regularization methods in Fig. 2 of the main text . . . 10
S3 Parameter-dependence of EN regularization error for Fig. 2 of the main text . . . 10
S4 Parameter-dependence of PGEN regularization error for Fig. 2 of the main text . . . 11
S5 L-curves for the regularization methods shown in Fig. 2 of the main text . . . 11
S6 Comparing L1 regularization with CVX and IRLS for different λ_1 . . . 12
S7 Exemplary traction fields reconstructed from noise-free artificial data . . . 13
S8 Exemplary traction fields reconstructed with Bayesian methods from artificial data with 5% noise . . . 14
S9 Exemplary comparison of error measures with artificial data using ten different methods . . . 15
S10 Comparison of various Bayesian methods using the real data from Fig. 5 of the main text . . . 15
S11 Evidence calculated with BL2 and ABL2 for the data shown in Fig. 5 of the main text . . . 16
S12 L-curve selection regularization parameter for the data shown in Fig. 5 of the main text . . . 16
S13 Selection of regularization parameters for the time sequence in Fig. 6 of the main text . . . 17

S14 Directed acyclic graphs representing tested complex Bayesian models (BCSL, BL and BEN) 17

1 Fast calculation of the matrix M using the shift theorem in Fourier space

Recently, it was suggested by Danuser and co-workers[1] that the computational effort for calculation of $\mathbf{M}$ can be significantly reduced by using the convolution theorem in Fourier space. In this section, we combine Fourier-space convolution with the shift theorem to calculate traction on a regular grid directly from irregularly spaced displacements. The starting point is a general linear relation between a continuous displacement field $U_i(\mathbf{x})$ and the traction field $F_j(\mathbf{x}')$. Both are two-dimensional vector fields with $(i,j) \in \{1,2\}$ and extend over the whole surface of the substrate Ω. They are related as[2]

$$U_i(\mathbf{x}) = \sum_{j=1}^{2} \int_\Omega G_{ij}(\mathbf{x}-\mathbf{x}')F_j(\mathbf{x}')\,\mathrm{d}^2\mathbf{x}'. \tag{1}$$

This equation is to be approximated as a matrix product. To discretize the traction field, we introduce a rectangular, regular mesh with meshsize w. The position of the mesh nodes is denoted by $\mathbf{y}_k$. The distance between any point in the traction field at $\mathbf{x}'$ and the mesh nodes at $\mathbf{y}_l$ is abbreviated for simplicity as $(d_1,d_2) = \mathbf{x}' - \mathbf{y}_l$. The traction at $\mathbf{x}'$ is assumed be a linear combination of the traction values at the four surrounding nodes. Hence, we introduce a pyramidal shape function, located at every node $\mathbf{y}_l$, which scales the traction magnitude at $\mathbf{x}'$ depending on the distances (d_1,d_2) as

$$\begin{aligned} h(d_1,d_2) =& \theta(d_1)\theta(d_2)(1-d_1/w)(1-d_2/w)\theta(w-d_1)\theta(w-d_2) + \theta(-d_1)\theta(d_2)(1+d_1/w)(1-d_2/w)\theta(w+d_1)\theta(w-d_2) \\ &+ \theta(d_1)\theta(-d_2)(1-d_1/w)(1+d_2/w)\theta(w-d_1)\theta(w+d_2) + \theta(-d_1)\theta(-d_2)(1+d_1/w)(1+d_2/w)\theta(w+d_1)\theta(w+d_2), \end{aligned}$$

where

$$\theta(x) := \begin{cases} 1, & x \geq 0 \\ 0, & x < 0 \end{cases}.$$

With the help of the shape function, the continuous traction field is linearly approximated as $F_j(\mathbf{x}') = \sum_l h(\mathbf{x}'-\mathbf{y}_l) f_{j,l}$. Thus, Eq. (1) becomes

$$U_i(\mathbf{x}) = \sum_{j,l} \int_\Omega G_{ij}(\mathbf{x}-\mathbf{x}')h(\mathbf{x}'-\mathbf{y}_l)\,\mathrm{d}^2\mathbf{x}'\, f_{j,l} = \sum_{j,l} \mathbf{M}'_{j,l}(\mathbf{x})\, f_{j,l}. \tag{2}$$

In order to simplify the convolution in Eq. (2) we employ a Fourier transformation with wave vector $\mathbf{k} = (k_1,k_2)$. The shape function becomes in Fourier space $\tilde{h}(k_1,k_2) = \mathrm{sinc}^2(k_1)\mathrm{sinc}^2(k_2)$ and the Green's function reads in Fourier space $\tilde{G}_{ij}(k_1,k_2) = 2(1+\nu)/(E(k_1^2+k_2^2)^{3/2})\left[(k_1^2+k_2^2)\delta_{ij} - k_i k_j \nu\right]$. On using the shift theorem, we find for Eq. (2)

$$U_i(\mathbf{x}) = \sum_{j,l} \mathrm{Ft}^{-1}(\tilde{G}_{ij}(\mathbf{k})\tilde{h}(\mathbf{k})e^{-i\mathbf{k}\mathbf{y}_l}) f_{j,l}. \tag{3}$$

The coefficient matrix $\mathbf{M}$ used in the main part of the paper is constructed from the $\mathbf{M}'_{j,l}(\mathbf{x})$ by inserting discrete measurement positions for $\mathbf{x}$ and arranging into matrix form.

2 Proximal gradient methods for L1 regularization (PGL) and EN regularization (PGEN)

Proximal gradient methods provide a way to robustly solve optimization problems involving locally non-differentiable, convex penalty functions. For our tests with TFM, we follow well-established approaches[3,4]. The target function to be considered for PGL and the PGEN reads

$$\frac{1}{2}||\mathbf{M}\mathscr{W}^*\mathscr{W}\mathbf{f}-\mathbf{u}||_2^2 + \lambda_1||\mathscr{W}\mathbf{f}||_1 + \frac{\lambda_2}{2}||\mathscr{W}\mathbf{f}||_2^2, \tag{4}$$

where $\mathscr{W}$ denotes an unitary wavelet transform. For wavelet transformation, we employ the lifting transform function provided with Ref.[5]. The non-differentiable penalty functions for L1 and EN regularization are denoted by $g(\mathbf{f}) = \lambda_1||\mathscr{W}\mathbf{f}||_1$ or $g(\mathbf{f}) = \lambda_1||\mathscr{W}\mathbf{f}||_1 + \lambda_2/2||\mathscr{W}\mathbf{f}||_2^2$, respectively. The optimization procedure is iterative and based on a gradient decent for the differentiable term $||\mathbf{M}\mathbf{f}-\mathbf{u}||_2^2/2$. If we were to employ a gradient decent for this term only, the traction at the iteration number $k+1$ would be given by $\mathbf{f}_{k+1} = \mathbf{h}_k$ with $\mathbf{h}_k = \mathbf{f}_k - \tau\mathbf{M}^T(\mathbf{M}\mathbf{f}_\mathbf{k} - \mathbf{u})$ where τ is the step size. However, since the solution must also obey the regularization constraints, the iteration is modified as follows

$$\mathbf{f}_{k+1} = \underset{\mathbf{f}}{\mathrm{argmin}}\{\tau g(\mathbf{f}) + \frac{1}{2}||\mathbf{h}_k - \mathbf{f}||^2\} =: \mathrm{prox}_{\tau g(\mathbf{f})}(\mathbf{h}_k). \tag{5}$$

Hence, the proximal gradient scheme produces incremental changes that balance a gradient decent to minimize the solution residual with a minimization of the penalty. The right-hand side of Eq. (5) is a definition of the so-called proximity operator. For the L1 norm, $g(x) = \lambda_1 |x|$, the proximity operator can be given in closed form as a threshold function to be applied to each element $\mathrm{prox}_{\tau\lambda_1 \|x\|_1}(x) = (|x| - \tau\lambda_1)_+ \mathrm{sgn}(x) =: S(x, \tau\lambda_1)$. Since the variables x are for our target function (4) the wavelet coefficients, result must be transformed back to real space after every iteration. The result is, see e.g.[3,4,6],

$$\mathbf{f}_{k+1} = \mathscr{W}^* S\left[\mathscr{W}\left(\mathbf{f}_k - \tau \mathbf{M}^\mathbf{T}(\mathbf{M}\mathbf{f}_k - \mathbf{u})\right); \tau\lambda_1\right]. \tag{6}$$

The iteration for the EN regularization in PGEN has the same structure, but an additional factor that shrinks the solution magnitude[4]

$$\mathbf{f}_{k+1} = \frac{1}{1+\tau\lambda_2} \mathscr{W}^* S\left[\mathscr{W}\left(\mathbf{f}_k - \tau \mathbf{M}^\mathbf{T}(\mathbf{M}\mathbf{f}_k - \mathbf{u})\right); \tau\lambda_1\right]. \tag{7}$$

In order to obtain a stable iteration, the step size needs to obey $\tau < 2/\|\mathbf{M}^\mathbf{T}\mathbf{M}\|_2$. Iterations are stopped when the change of the solution norm from timestep to timestep is smaller than $10^{-8}\%$.

3 Bayesian compressive sensing using Laplace prior (BCSL)

To test an example of a more elaborate Bayesian hierarchical model for TFM, we employ a software representing the network shown in Fig. S14(a). The system is described in detail in Ref.[7]. A main difference between this algorithm and ABL2 is the assumption of sparsity through a Laplace prior that is employed along with further hyperpriors for the noise. The model is solved with a variational technique. As shown in the Supplementary figures, the algorithm effectively produces a sparse image, however, with strongly overestimated traction hotspots. Addition of noise to the displacement field leads to a very visible degradation of the reconstruction quality with many false traction hotspots appearing.

4 Bayesian Lasso (BL) and Bayesian elastic net (BEN)

As an alternative to the variational Bayes approach, hierarchical models can be solved by Markov chain Monte Carlo methods. To test this approach in conjunction with complex models, we employ two models provided as Matlab packages together with Ref.[8]. The BL is based on the same network structure as the BCSL shown in Fig. S14(a). Furthermore, we also tested a Bayesian version of the elastic net (BEN) where the network is shown in Fig. S14(b). Both algorithms perform similarly to the BCSL for very low noise. Here, the assumption of sparsity helps to produce a clear background and allows to distinguish traction sites clearly. However, traction magnitude estimates are strongly exaggerated. As expected, the full solution of the models via Monte Carlo sampling makes the performance of BL and BEN slightly more robust than BCSL in the presence of noise. Overall, the performance of these methods in the context of TFM is unsatisfactory.

5 Analytical calculation of displacements around a circular traction patch

For convenience of the reader, we provide here explicit formulas for the displacement field around a single circular traction patch. Test images containing multiple patches can be assembled by adding the displacement vectors resulting from the individual patches. The analytical solution is calculated for a patch with radius R located at the origin. We employ polar coordinates, $\mathbf{r} = (r\cos\theta, r\sin\theta)$, with r and θ being the radial and angular coordinate. The traction patch is described as

$$f(r) = \begin{cases} f_0, & |r| < R, \\ 0, & |r| \geq R. \end{cases} \tag{8}$$

The traction vector is given by $f_x = f(r)\cos\gamma$ and $f_y = f(r)\sin\gamma$, where γ is the angle between the x axis and the direction of the traction. Fourier transformation yields

$$\tilde{f}(\rho) = \int_0^{2\pi}\int_0^{\infty} f(r) e^{i\rho r\cos(\phi-\theta)} r\mathrm{d}r\mathrm{d}\theta = 2\pi R f_0 J_1(\rho R)/\rho, \tag{9}$$

where $J_1(\rho R)$ is a Bessel function, ρ is a radial wave vector, and ϕ is an angle. The traction vector in Fourier space is thus $\tilde{f}_x = \tilde{f}(\rho)\cos\gamma$ and $\tilde{f}_y = \tilde{f}(\rho)\sin\gamma$. The Fourier space Green's function in polar coordinates reads

$$\tilde{G}_{ij}(\rho,\phi) = \frac{2(1+\nu)}{E\rho}\begin{bmatrix} (1-\nu)+\nu\sin^2\phi & -\nu\sin\phi\cos\phi \\ -\nu\sin\phi\cos\phi & (1-\nu)+\nu\cos^2\phi \end{bmatrix}. \tag{10}$$

According to the convolution theorem, the displacement field in Fourier space becomes

$$\begin{bmatrix}\tilde{u}_x\\ \tilde{u}_y\end{bmatrix} = \frac{2(1+\nu)}{E\rho}\begin{bmatrix}(1-\nu)+\nu\sin^2\phi & -\nu\sin\phi\cos\phi\\ -\nu\sin\phi\cos\phi & (1-\nu)+\nu\cos^2\phi\end{bmatrix}\begin{bmatrix}\tilde{f}_x\\ \tilde{f}_y\end{bmatrix}. \tag{11}$$

Thus, displacements in real space can be calculated through inverse Fourier transformation

$$\begin{bmatrix}u_x\\ u_y\end{bmatrix} = \left(\frac{1}{2\pi}\right)^2\int_0^{2\pi}\int_0^{\infty}\begin{bmatrix}\tilde{u}_x\\ \tilde{u}_y\end{bmatrix} e^{-i\rho r\cos(\phi-\theta)}\rho \mathrm{d}\rho \mathrm{d}\phi. \tag{12}$$

The result can be simplified as

$$\begin{aligned} u_x(r,\theta) &= \frac{R(1+\nu)}{\pi E}\left[\Big((1-\nu)N_1+\nu N_2\Big)f_0\cos\gamma - \nu N_3 f_0\sin\gamma\right],\\ u_y(r,\theta) &= \frac{R(1+\nu)}{\pi E}\left[-\nu N_3 f_0\cos\gamma + \Big((1-\nu)N_1+\nu N_4\Big)f_0\sin\gamma\right]. \end{aligned} \tag{13}$$

For the functions N_1 to N_4 we have for the inner region where $r < R$ and $\xi_1 = r^2/R^2$

$$N_1 = 4E_0(\xi_1) \tag{14a}$$

$$N_2 = \frac{4\cos(2\theta)\Big((r^2+R^2)E_0(\xi_1)+(r^2-R^2)K_0(\xi_)\Big)}{3r^2} + 4\sin^2\theta E_0(\xi_1) \tag{14b}$$

$$N_3 = \frac{2\sin(2\theta)\Big((r^2-2R^2)E_0(\xi_1)+2(R^2-r^2)K_0(\xi_1)\Big)}{3r^2} \tag{14c}$$

$$N_4 = 4\cos^2\theta E_0(\xi_1) - \frac{4\cos(2\theta)\Big((r^2+R^2)E_0(\xi_1)+(r^2-R^2)K_0(\xi_1)\Big)}{3r^2}. \tag{14d}$$

Here, E_0 is complete elliptic integral of the first kind and K_0 is complete elliptic integral of the second kind. For the outer region where $r > R$ and $\xi_2 = R^2/r^2$ we have

$$N_1 = \frac{4\Big(r^2E_0(\xi_2)+(R^2-r^2)K_0(\xi_2)\Big)}{rR} \tag{15a}$$

$$N_2 = \frac{\Big(6r^2-2(r^2-2R^2)\cos(2\theta)\Big)E_0(\xi_2)+2(r^2-R^2)(\cos(2\theta)-3)K_0(\xi_2)}{3rR} \tag{15b}$$

$$N_3 = \frac{2\sin(2\theta)\Big((r^2-2R^2)E_0(\xi_2)+(R^2-r^2)K_0(\xi_2)\Big)}{3rR} \tag{15c}$$

$$N_4 = \frac{\Big(6r^2+2(r^2-2R^2)\cos(2\theta)\Big)E_0(\xi_2)-2(r^2-R^2)(\cos(2\theta)+3)K_0(\xi_2)}{3rR}. \tag{15d}$$

6 Implementation of the regularization routines

For L2 regularization in real space we employ a singular value composition and the routines "tikhonov" and "l_curve" provided by the Matlab package "Regularization Tools".[9] The use of this package for TFM has been described earlier.[10,11]
To perform L1- and EN regularization we minimize well-established formulas[12] using the convex optimization package CVX.[13,14] The target functions for L1 regularization and EN regularization are given by

$$\hat{\mathbf{f}} = \underset{\mathbf{f}}{\operatorname{argmin}}\left[\mathbf{f}^{\mathrm{T}}\mathbf{M}^{\mathrm{T}}\mathbf{M}\mathbf{f} - 2\mathbf{u}^{\mathrm{T}}\mathbf{M}\mathbf{f} + \mathbf{f}^{\mathrm{T}}\mathbf{f} + \lambda_1\|\mathbf{f}\|_1\right], \tag{16}$$

$$\hat{\mathbf{f}} = \underset{\mathbf{f}}{\operatorname{argmin}}\left[\mathbf{f}^{\mathrm{T}}\left(\frac{\mathbf{M}^{\mathrm{T}}\mathbf{M}+\lambda_2\mathbf{I}}{1+\lambda_2}\right)\mathbf{f} - 2\mathbf{u}^{\mathrm{T}}\mathbf{M}\mathbf{f} + \lambda_1\|\mathbf{f}\|_1\right]. \tag{17}$$

Comparison of the two formulas shows that EN regularization is a stabilized version of the L1 regularization. Below, we provide a short Matlab code for L1- and EN regularization.

```
function F = L1_EN_cvx(n,X,u,lambda1,lambda2)
% This code requires the convex optimization package CVX.
% n is the lenght of the traction vector "f"; X the matrix "M" in the main text;
% u is the displacement vector; lambda1 and lambda2 are regularization parameters.

a = size(X);
R= eye(a(2))

cvx_begin
cvx_solver sedumi;
variable f(n);

% This line is for L1 regularization. Comment out if not needed.
minimize((X*f-u)'*(X*f-u)+lambda1*(norm(f,1)));

% This line is for EN regularization. Comment out if not needed.
minimize(f'*(X'*X+lambda2*R)/(1+lambda2)*f-2*u'*X*f+lambda1*(norm(f,1)));

cvx_end;
end
```

7 L1 regularization using Iterative Reweighted Least Squares (IRLS)

The L1 regularization problem $\hat{\mathbf{f}} = \underset{\mathbf{f}}{\operatorname{argmin}}\left[\|\mathbf{Mf}-\mathbf{u}\|_2 + \lambda_1\|\mathbf{f}\|_1\right]$ can be solved with different approaches. As an alternative to the popular convex optimization, we tested the iteratively reweighted least squares algorithm (IRLS).[15–17] This algorithm approximates the result at every iteration i as

$$\mathbf{f}^{i+1} = \underset{\mathbf{f}^{i+1}}{\operatorname{argmin}}\left[\|\sum_{k=1}^{2n}\sum_{l=1}^{2m} M_{lk} f_k^{i+1} - u_l\|_2 + \frac{\lambda_1}{2}\sum_{k=1}^{2n}\frac{|f_k^{i+1}|^2}{|f_k^i|}\right]. \tag{18}$$

As initial condition for this scheme we chose $\mathbf{f}^{i=0} = \mathbf{1}$. The IRLS can be seen as a reweighted, iterative L2 scheme. In our tests, this method exhibited favorable properties with less pronounced over-estimation of local traction.

8 Implementation of Bayesian TFM routines

The code for Bayesian TFM includes standardization of the data, finding the optimal regularization parameters, solving the regularized problem, and finally undoing the standardization. For regularization in BL2 and ABL2 we employ the Matlab package "Regularization Tools" that requires a singular value decomposition of the problem.[9] Below, we provide an exemplary code for BL2.

```
function F = reconstruction_BL2(X, u, noise_u)
% This code requires the Matlab package ``Regularization Tools'' by P.C. Hansen.
% X the matrix "M" in the main text; u is the displacement vector;
% noise_u is a vector with displacement noise recorded far away from cells.
% The output is a vector with traction forces.

%calculate inverse noise variance
beta = 1/var(noise_u);

% standardize the input data
sd = std(X);
X= (X-repmat(mean(X,1),size(X,1),1))./repmat(std(X),size(X,1),1);
u = u-mean(u);
```

```
% singlular value decomposition of X
[U,s,V] = csvd(X);

XX=X'*X;
aa=size(X);
c=ones(aa(2),1); C=diag(c);

% parameters for Golden Section search to find maximum of log evidence
alpha1 = 200;        % initial left alpha
alpha2 = 40000;      % initial right alpha
step_size = 30;      % step size
max_n = 200;         % iteration number

% Golden Section search to find maximum of log evidence
n  = 0; nn = []; lambda = [];
 while 1
    middle = 0.5*(alpha1+alpha2);
    middle_up = middle + 0.5*step_size;
    evidence_up = logevidence(middle_up,beta,U,s,V,u,C,X,aa,XX);

    middle_down = middle - 0.5*step_size;
    evidence_down = logevidence(middle_down,beta,U,s,V,u,C,X,aa,XX);

     if evidence_up >= evidence_down
        alpha1 = middle_down;
     else
        alpha2 = middle_up;
     end

     n=n+1;
     nn(n)=n;
     lambda(n) = alpha1/beta;

     if norm(alpha1-alpha2)/norm(alpha1) < 1e-5 || n == max_n
      break
     end
  end

% calculation of final solution with optimal parameters
 [F] = tikhonov(U,s,V,u,lambda_2);

% undo standardization
  F = F./sd';
end

%%% subroutine for calculating the log evidence function
function evidence_value= logevidence(falpha,fbeta,U,s,V,fu,fC,X,aa,XX)

        flambda = falpha/fbeta;
        [F] = tikhonov(U,s,V,fu,flambda);

        % calculate log(det(A))
        A = falpha*fC+fbeta*XX;
        L = chol(A);
```

```
    logdetA = 2*sum(log(diag(L)));

    % formula for log evidence
    evidence_value = -0.5*falpha*F'*F - 0.5*fbeta*(X*F-fu)'*(X*F-fu) ...
            -0.5*logdetA + 0.5*aa(1)*log(fbeta) + 0.5*aa(2)*log(falpha)...
            -0.5*aa(1)*log(2*pi);
end
```

References

1. Han, S. J., Oak, Y., Groisman, A. & Danuser, G. Traction microscopy to identify force modulation in subresolution adhesions. *Nat. Methods* **12**, 653–656 (2015).
2. Landau, L. D. & Lifshitz, E. Theory of elasticity, vol. 7. *Course Theor. Phys.* **3**, 109 (1986).
3. Beck, A. & Teboulle, M. A fast iterative shrinkage-thresholding algorithm with application to wavelet-based image deblurring. In *ICASSP 2009. IEEE International Conference on Acoustics, Speech and Signal Processing.*, 693–696 (IEEE, 2009).
4. Parikh, N., Boyd, S. *et al.* Proximal algorithms. *Foundations Trends Optim.* **1**, 127–239 (2014).
5. Peyré, G. The numerical tours of signal processing. *Comput. Sci. Eng.* **13**, 94–97 (2011).
6. Figueiredo, M. A. & Nowak, R. D. An em algorithm for wavelet-based image restoration. *IEEE Transactions on Image Process.* **12**, 906–916 (2003).
7. Babacan, S. D., Molina, R. & Katsaggelos, A. K. Bayesian compressive sensing using laplace priors. *IEEE Trans. Image Process.* **19**, 53–63 (2010).
8. Korobilis, D. Hierarchical shrinkage priors for dynamic regressions with many predictors. *Int. J. Forecast.* **29**, 43–59 (2013).
9. Hansen, P. C. Regularization tools version 4.0 for matlab 7.3. *Numer. algorithms* **46**, 189–194 (2007).
10. Schwarz, U. S. *et al.* Calculation of forces at focal adhesions from elastic substrate data: the effect of localized force and the need for regularization. *Biophys. J.* **83**, 1380–1394 (2002).
11. Sabass, B., Gardel, M. L., Waterman, C. M. & Schwarz, U. S. High resolution traction force microscopy based on experimental and computational advances. *Biophys. J.* **94**, 207–220 (2008).
12. Zou, H. & Hastie, T. Regularization and variable selection via the elastic net. *J. R. Stat. Soc. Ser. B Stat. Methodol.* **67**, 301–320 (2005).
13. Grant, M. & Boyd, S. CVX: Matlab software for disciplined convex programming, version 2.1. http://cvxr.com/cvx (2014).
14. Grant, M. & Boyd, S. Graph implementations for nonsmooth convex programs. In Blondel, V., Boyd, S. & Kimura, H. (eds.) *Recent Advances in Learning and Control*, Lecture Notes in Control and Information Sciences, 95–110 (Springer-Verlag Limited, 2008). http://stanford.edu/~boyd/graph_dcp.html.
15. van Wieringen, W. N. Lecture notes on ridge regression. *arXiv preprint arXiv:1509.09169* (2015).
16. Scales, J. A., Gersztenkorn, A. & Treitel, S. Fast ip solution of large, sparse, linear systems: Application to seismic travel time tomography. *J. Comput. Phys.* **75**, 314–333 (1988).
17. Suñé-Auñón, A. *et al.* Full l 1-regularized traction force microscopy over whole cells. *BMC Bioinf.* **18**, 365 (2017).

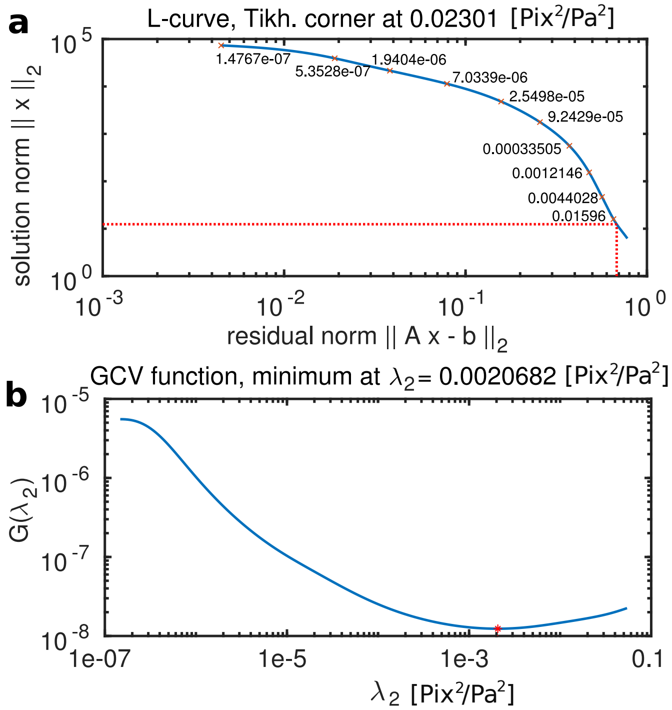

Figure S1. **Classical methods for selecting the regularization parameter λ_2 with the L-curve and GCV for strong noise $\sigma_{\mathbf{n}}/\sigma_{\bar{\mathbf{u}}} \simeq 0.85$** The L2 regularization parameters suggested by the L-curve criterion and the GCV differ considerably, about by a factor of ten. Data is artificial and consists of 15 circulars traction spots.

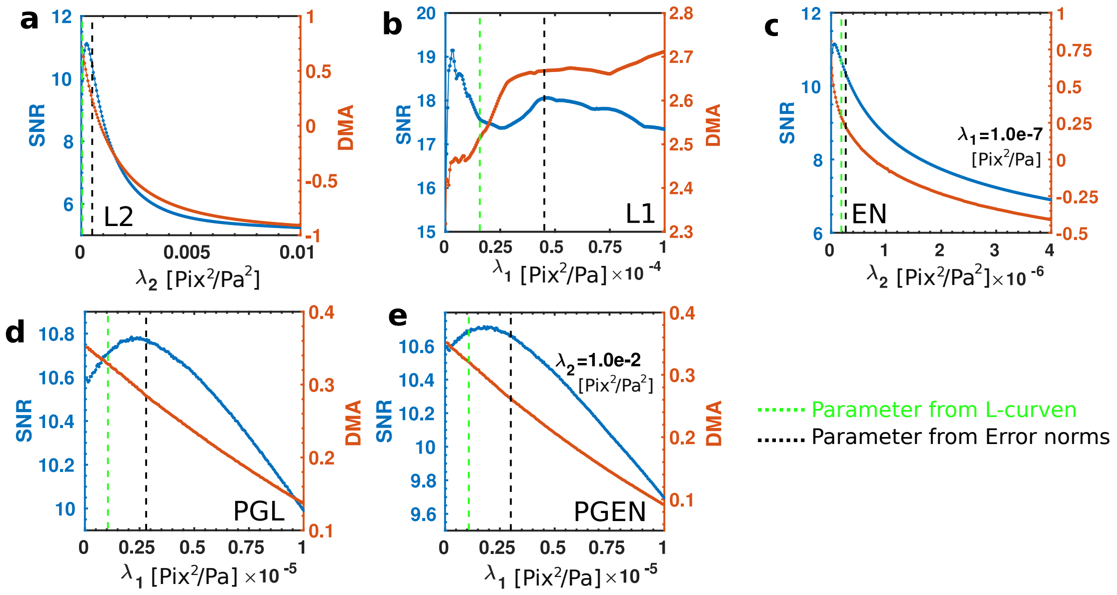

Figure S2. Additional error quantification for the regularization examples shown in Fig. 2 of the main text. Figures (a)-(e) are the signal to noise ratios (SNR) and deviations of traction maxixima (DMA) for the same tests as shown in Fig. 2(c) of the main text.

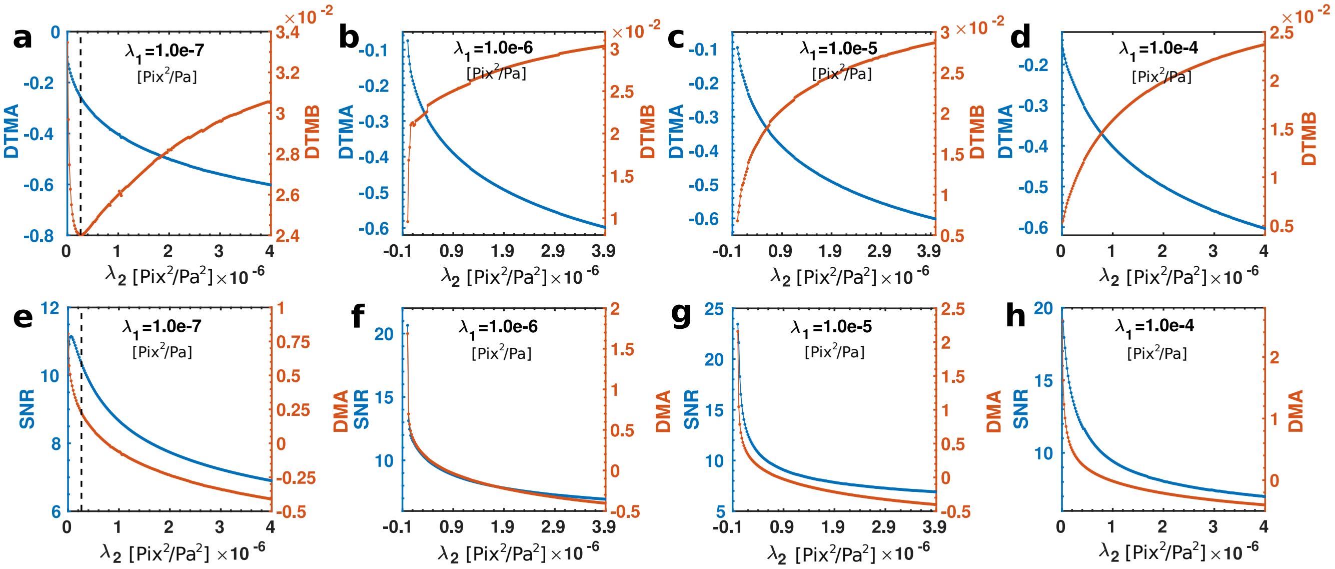

Figure S3. Parameter-dependence of EN regularization error at fixed values of λ_1. (a)-(d) The DTMA and DTMB. (e)-(h) SNR and DTM.

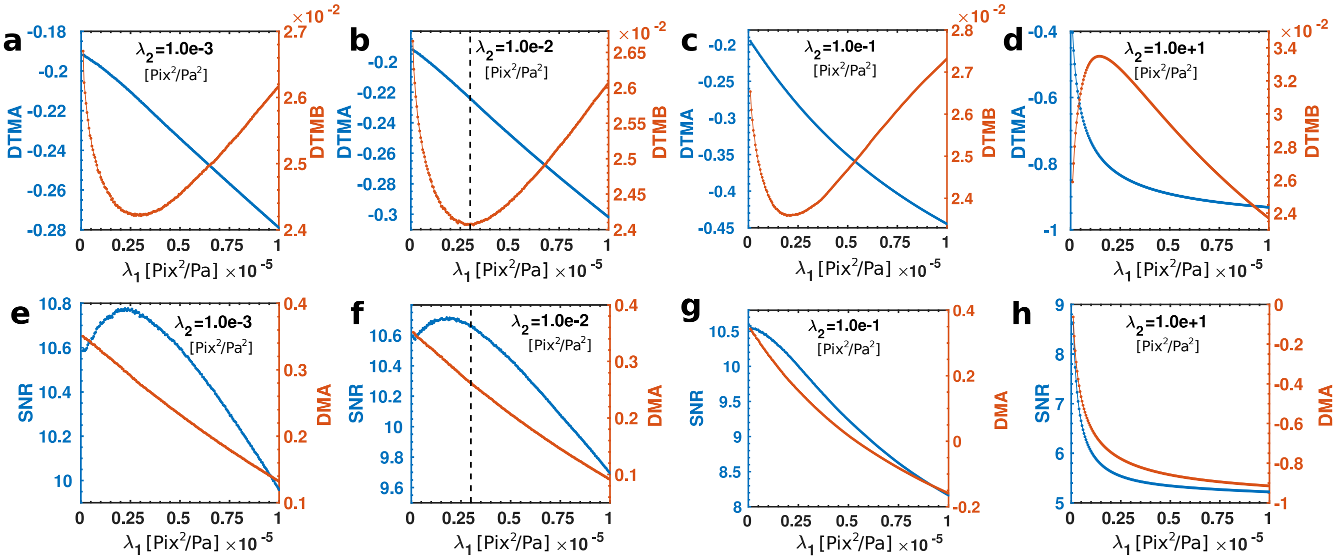

Figure S4. Parameter-dependence of PGEN regularization error at fixed values of λ_2. (a)-(d) Error norms DTMA and DTMB. (e)-(h) SNR and DTM.

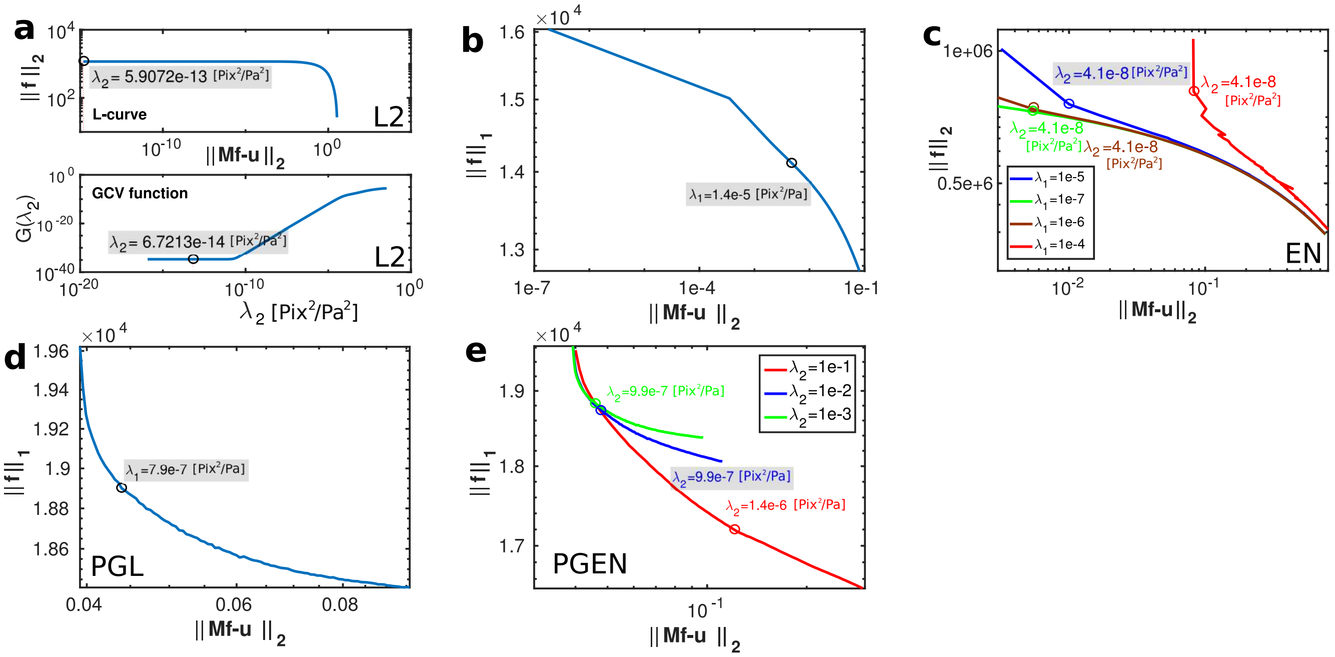

Figure S5. L-curves for the regularization methods shown in Fig. 2 of the main text. (a) L-curve and GCV function for the classical L2 regularization. (b)-(c) L-curves for L1- and EN-regularization. (d)-(e) L-curves for PGL and PGEN.

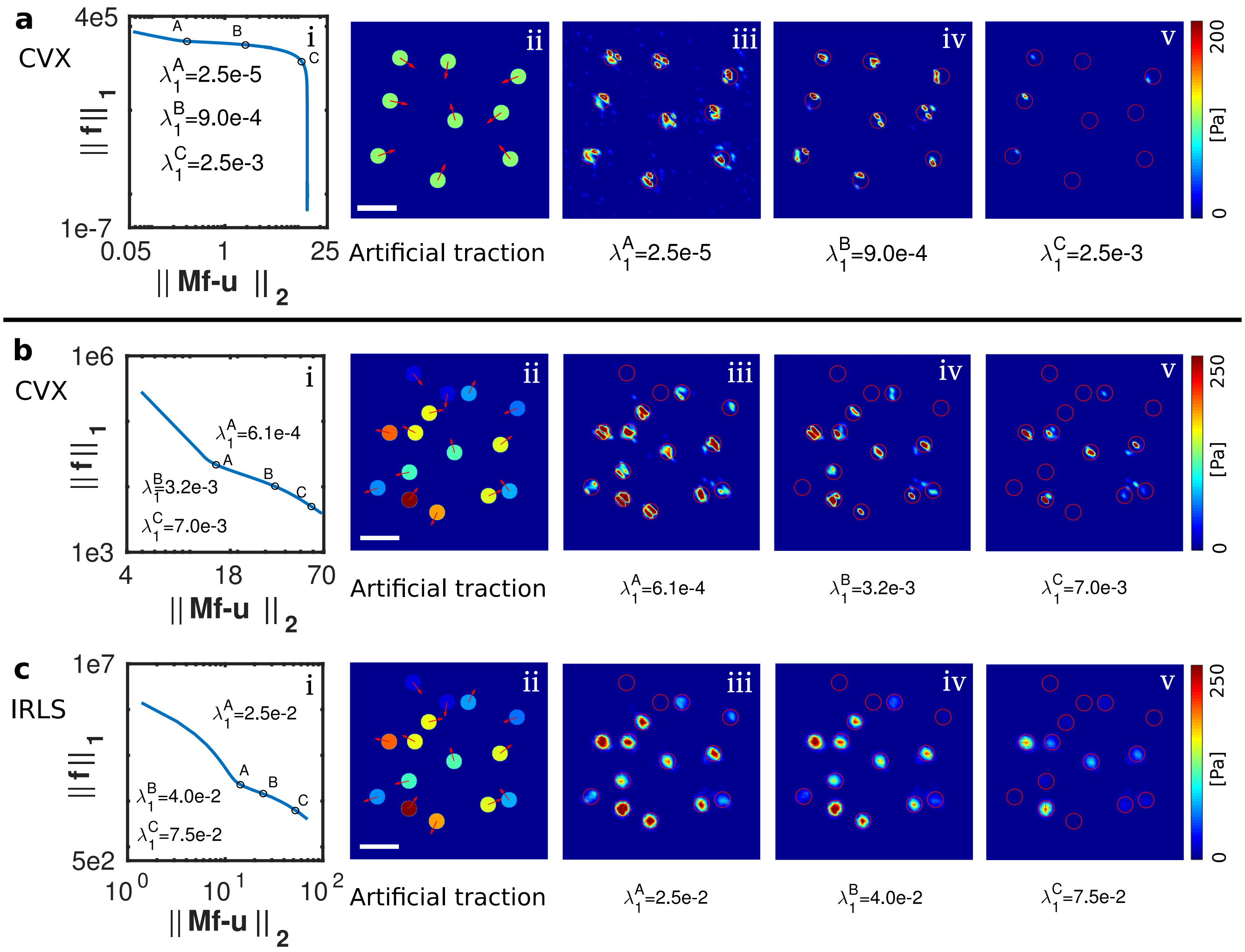

Figure S6. Comparison of results of L1 regularization using CVX and IRLS for different λ_1. See Fig. 3 of the main text. (a) Artificial data with traction spots having all the same magnitude. Displacements are corrupted with 2% noise. (a-i) The L-curve exhibits a turning point indicating a transition from a data-dominated to a regularization-dominated regime. (a-iii)-(a-v) Traction fields obtained for λ_1^A, λ_1^B and λ_1^C. (b) Artificial data with traction spots having different magnitudes and 2% displacement noise. (b-iii)-(b-v) Traction reconstruction with CVX. (c) Same data as before, but regularization done with IRLS. For all samples, the regularization parameter should be chosen well above the turning point of the L-curve to avoid partial suppression of traction patterns. In our experience, the IRLS produces more accurate results than the L1 regularization with CVX.

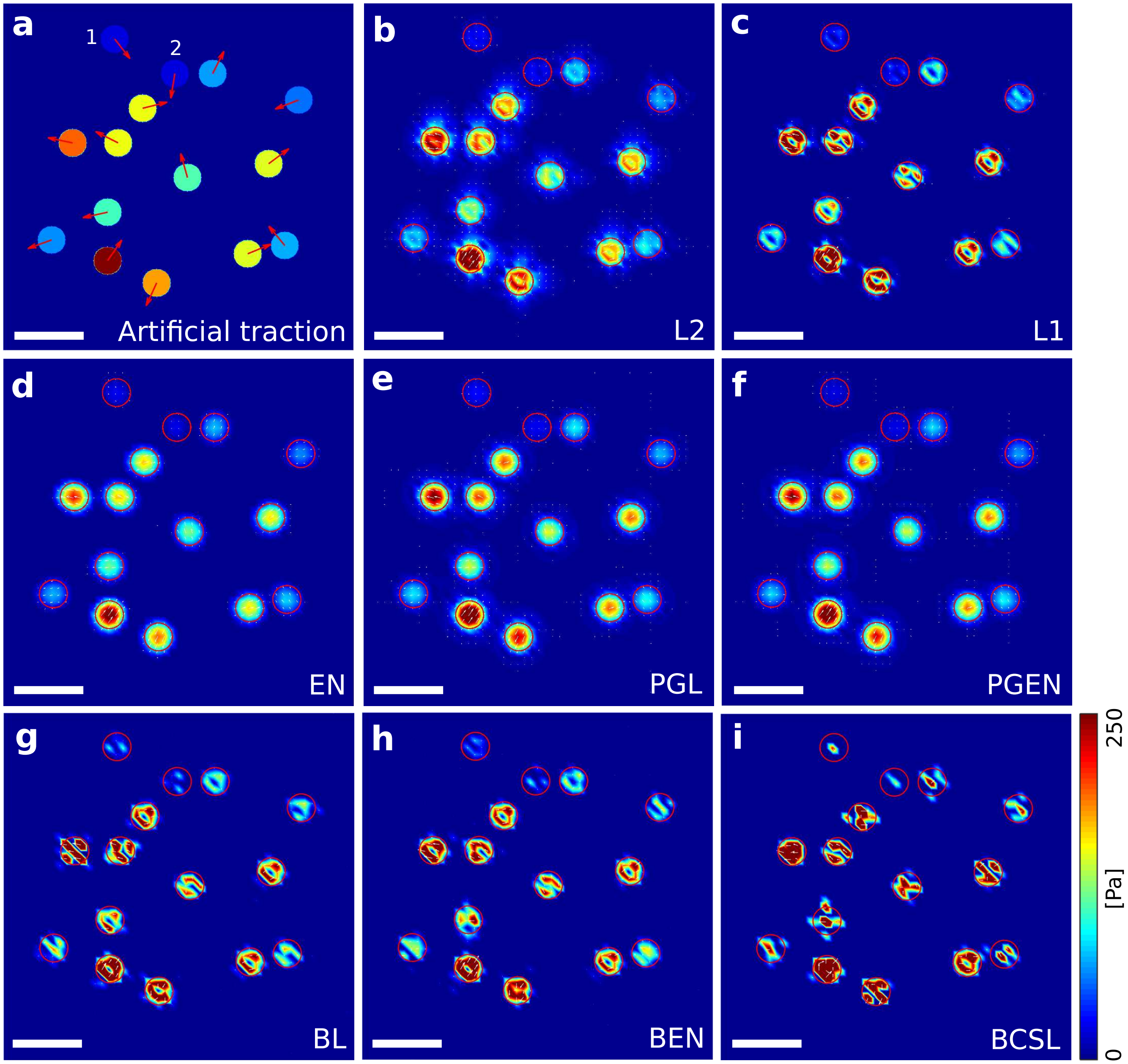

Figure S7. Exemplary traction fields reconstructed from noise-free artificial data. (a) The artificial data consists of 15 circular traction patterns with random magnitude (from 0-250 Pa). (b)-(f) Reconstructed traction fields obtained with the regularization methods L2, L1, EN, PGL and PGEN, respectively. (**g**)-(**i**) Reconstructed traction fields obtained using the complex Bayesian hierarchical network algorithms BL, BEN and BCSL. Space bar 5 μm.

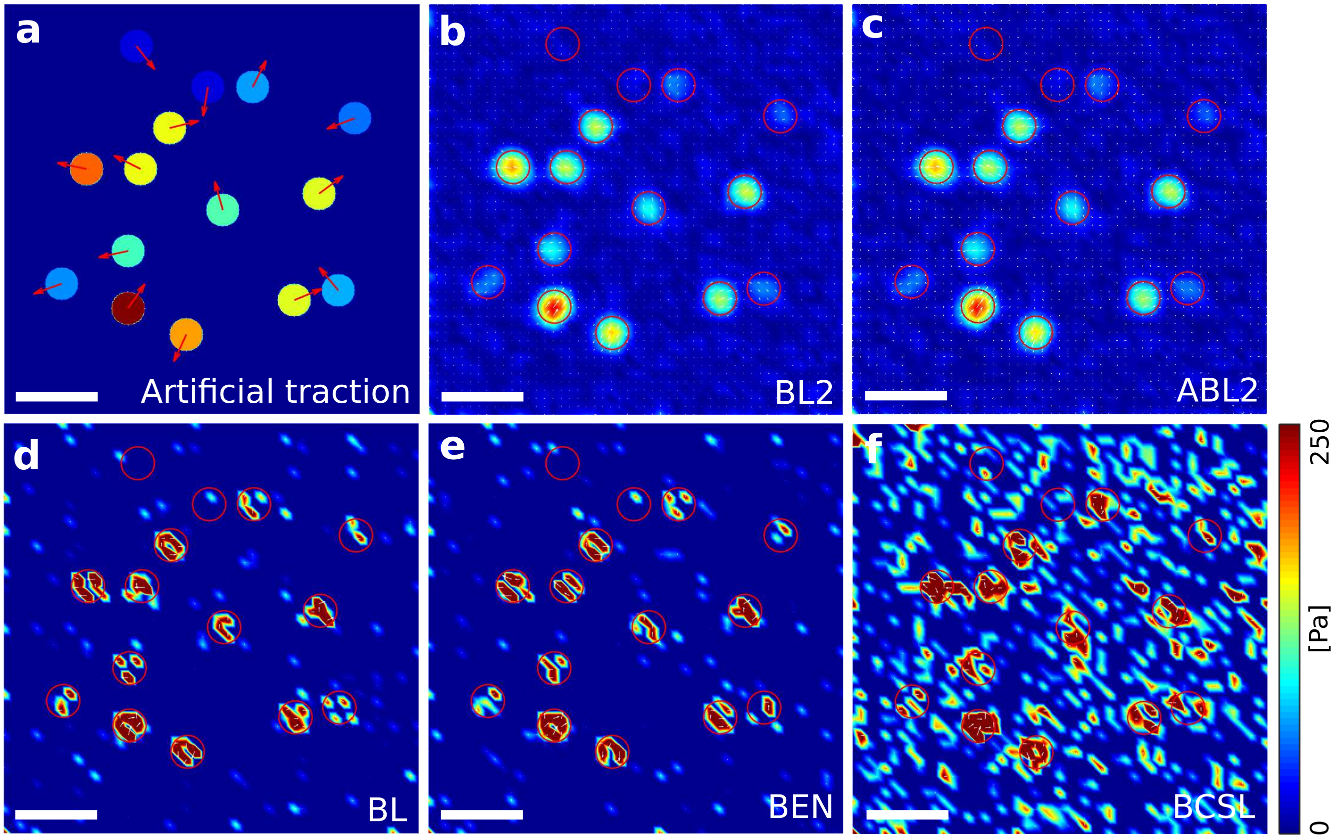

Figure S8. **Exemplary traction fields reconstructed with Bayesian methods from artificial data containing 5% noise.** (a) Artificial data as in Fig. S5 (a), but 5% Gaussian noise is added to the displacements. (b)-(c) Reconstructed traction from BL2 and ABL2, respectively. (d)-(f) Reconstruction traction using the Bayesian hierarchical network algorithms BL, BEN and BCSL, respectively. Space bar 5 μm.

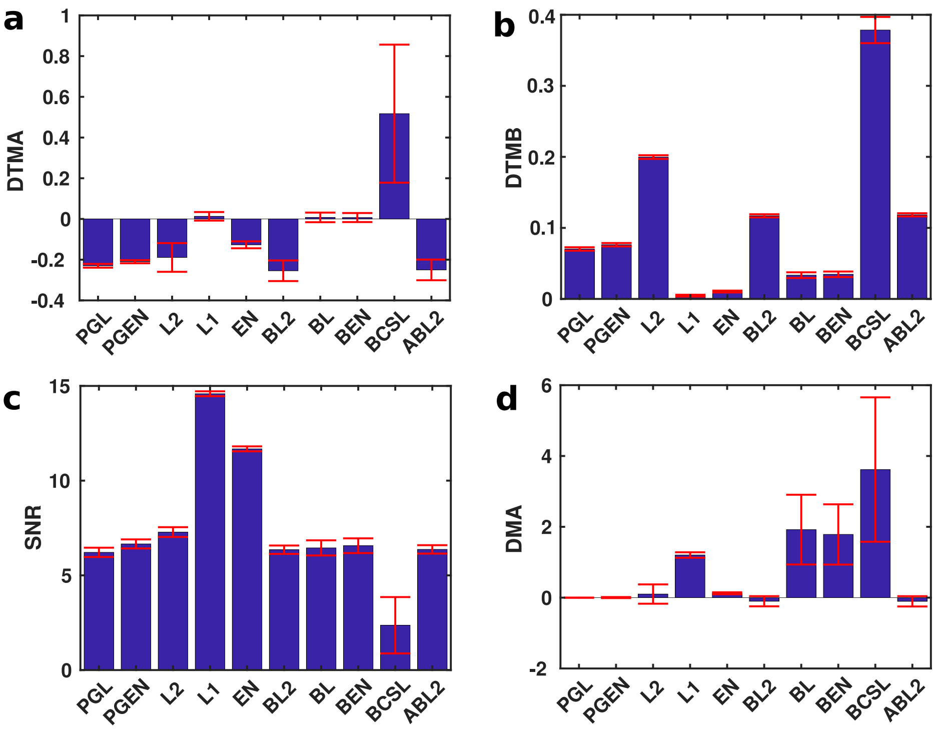

Figure S9. Exemplary comparison of errors of ten different methods employed for reconstruction of artificial data with 5% noise. (a) DTMA with error bars, s.d. (standard deviation error bars). (b) DTMB with error bars, s.e.m.(standard error of the mean). (c) SNR with error bars, s.e.m. (d) DMA with error bars, s.d.

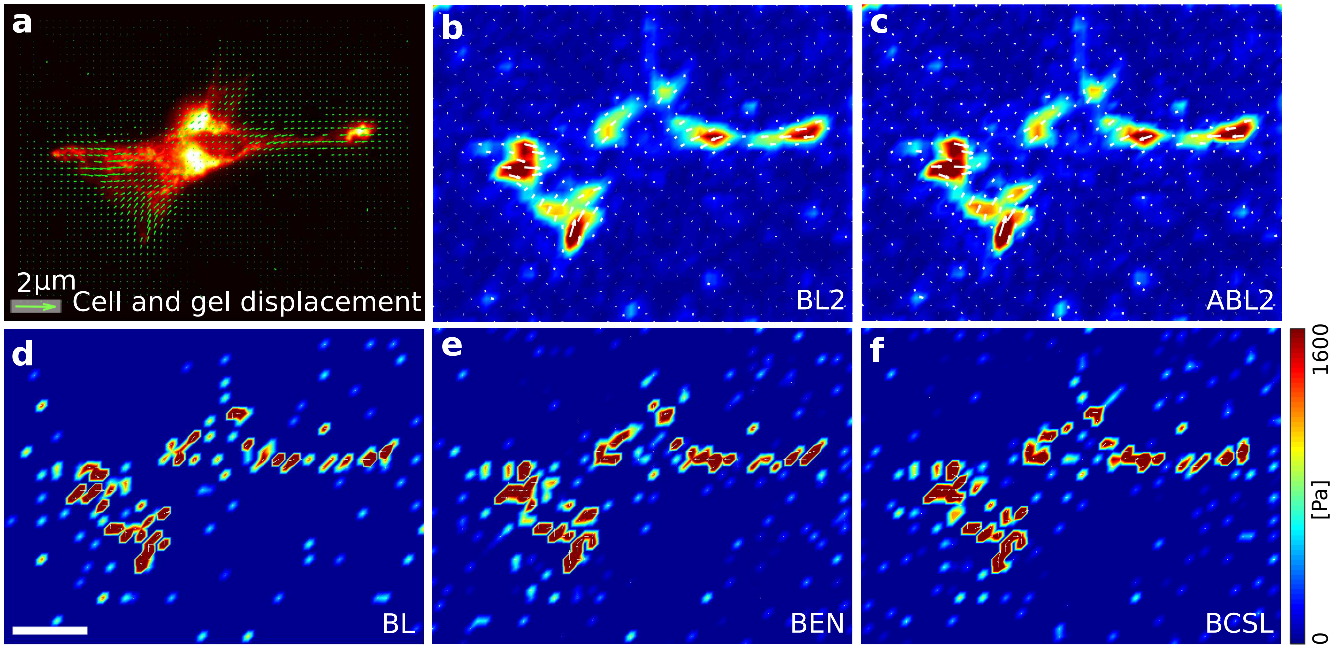

Figure S10. Comparison of various Bayesian methods for TFM. (a) Image of cell and displacement field (Green vectors). (b)-(c) Reconstruction using BL2 and ABL2, respectively.(d)-(f) The hierarchical Bayesian network algorithms BL, BEN, and BCSL produce very sparse patterns with strongly overestimated traction. Space bar 25 μm.

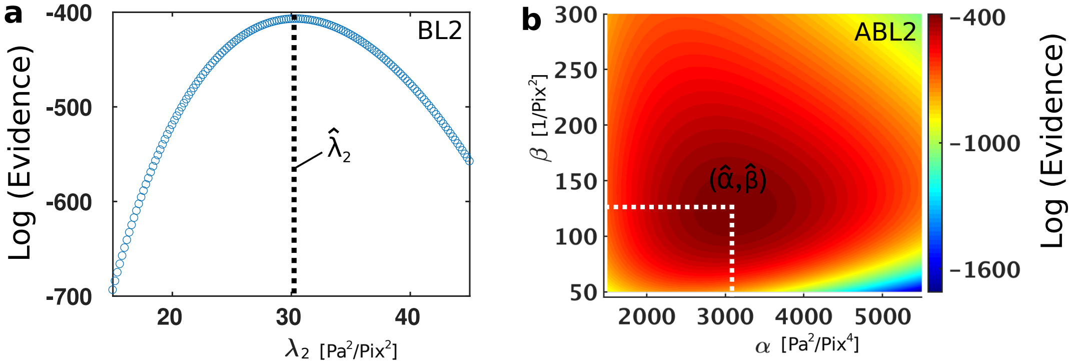

Figure S11. Plot of the logarithmic evidence functions calculated with BL2 and ABL2 for the data shown in Fig. 5 of the main text. (a) The logarithmic evidence curve calculated in BL2 as function of the regularization parameter. The variance of the measurement noise is estimated from the data to be about $0.01\,\mathrm{Pix}^2$. (b) Map of the logarithmic evidence for ABL2. The maximum of this function is located at $1/\beta = 0.008\,\mathrm{Pix}^2$, which is close to the noise variance estimated from the data.

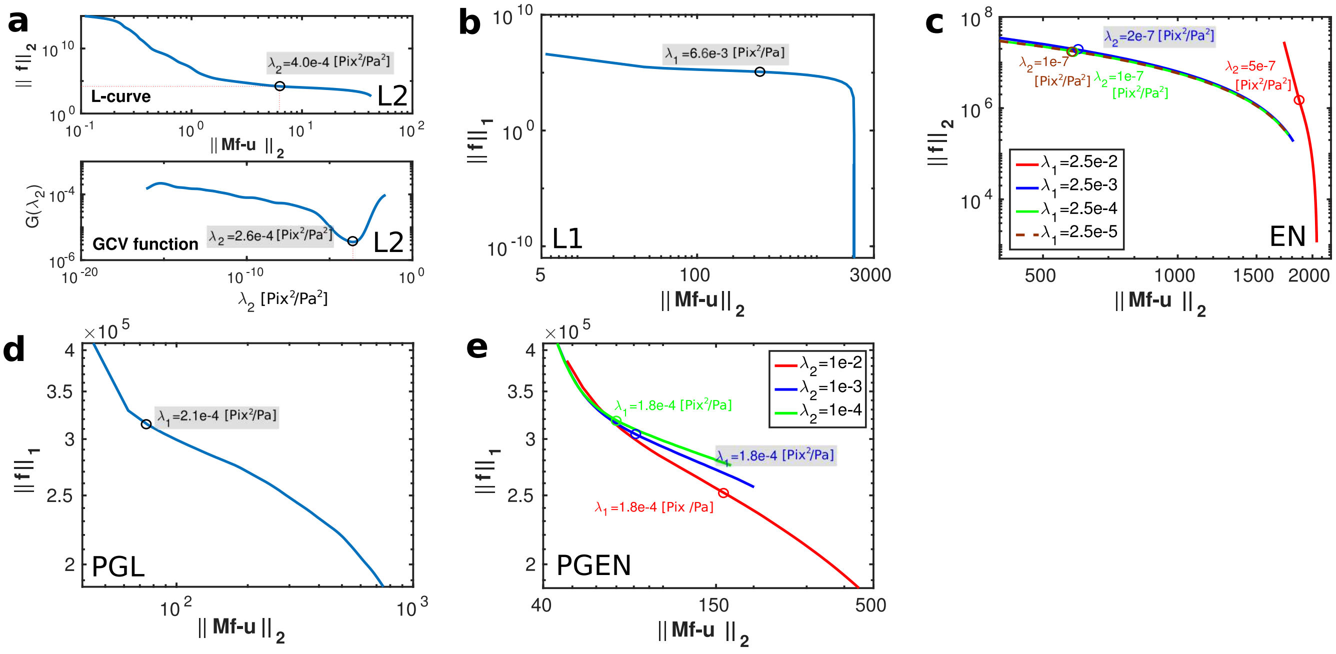

Figure S12. Regularization parameter selection by L-curve for the data shown in Fig. 5 of the main text. (a) L-curve and GCV function for the classical L2 regularization. (b)-(c) L-curves for L1- and EN-regularization. (d)-(e) L-curves for PGL and PGEN.

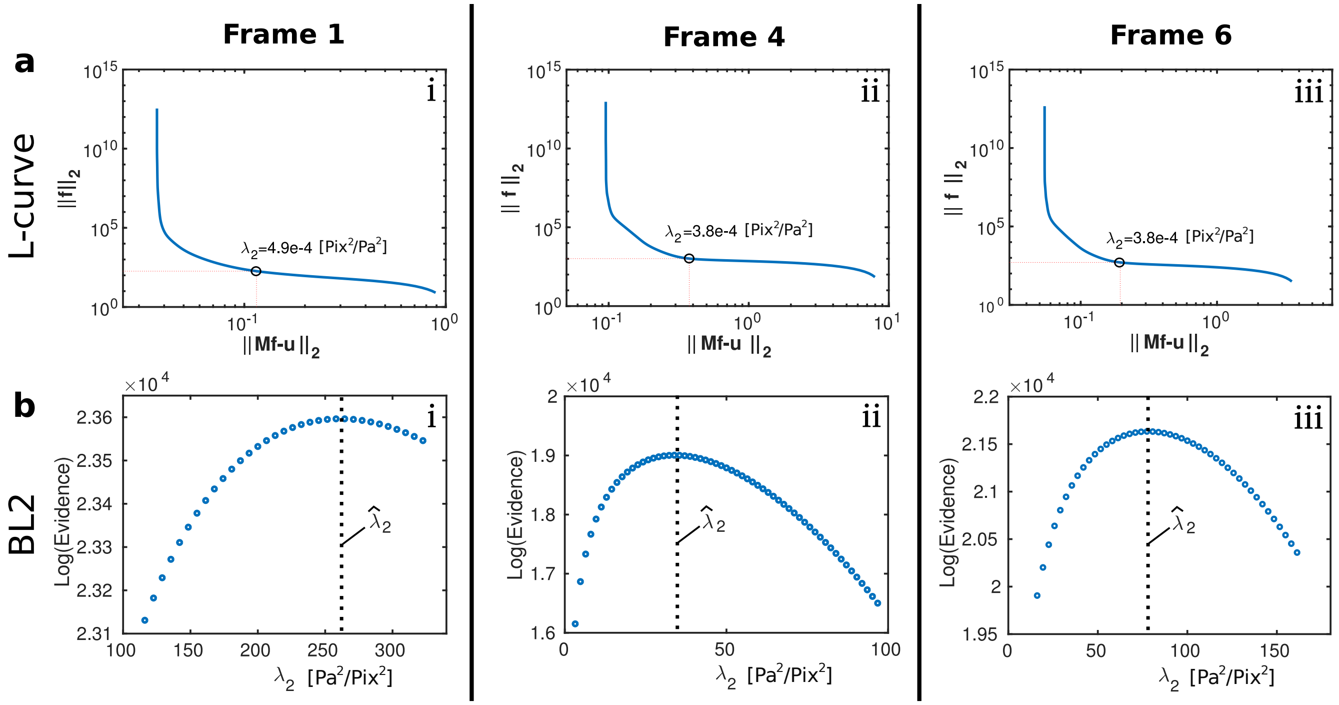

Figure S13. **Selection of regularization parameters for the three frames of the time sequence in shown Fig. 6 of the main text.** (a-i)-(a-iii) L-curves for the choice of parameters used with standard L2 regularization. (b-i)-(b-iii) The evidence functions calculated with BL2 show very clear maxima that are localized automatically for optimal regularization.

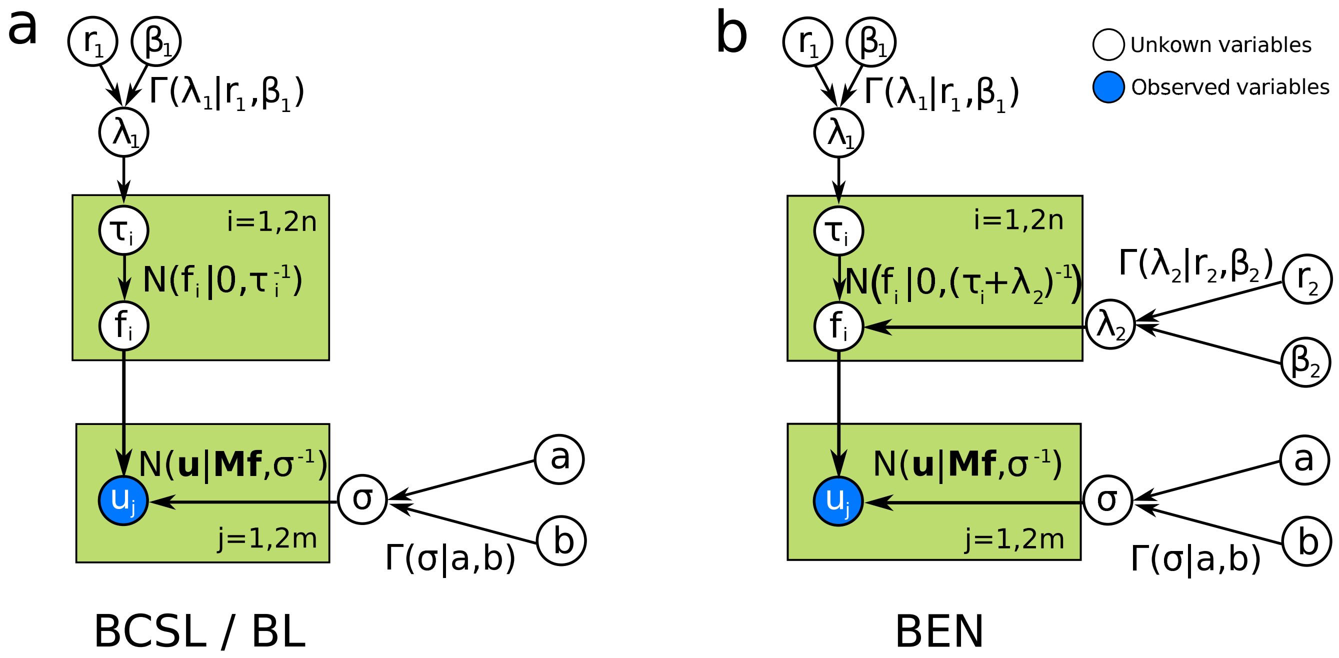

Figure S14. **Directed acyclic graphs representing the complex Bayesian models (BCSL, BL and BEN) that have been tested with this work.** Some test results using these methods for TFM are shown in the supplementary Fig. S8.
